# Supplementary material for: The impact of aztreonam–clavulanic acid exposure on gene expression and mutant selection using a multidrug-resistant E. coli
Source: Microbiol Spectr. 2025 Feb 11;13(3):e01782-24. doi: 10.1128/spectrum.01782-24 (PMC11878011; doi:10.1128/spectrum.01782-24)
Supplement: Supplemental Figures — Figures S1 to S6. [file spectrum.01782-24-s0001.pdf]

Supplementary Figures

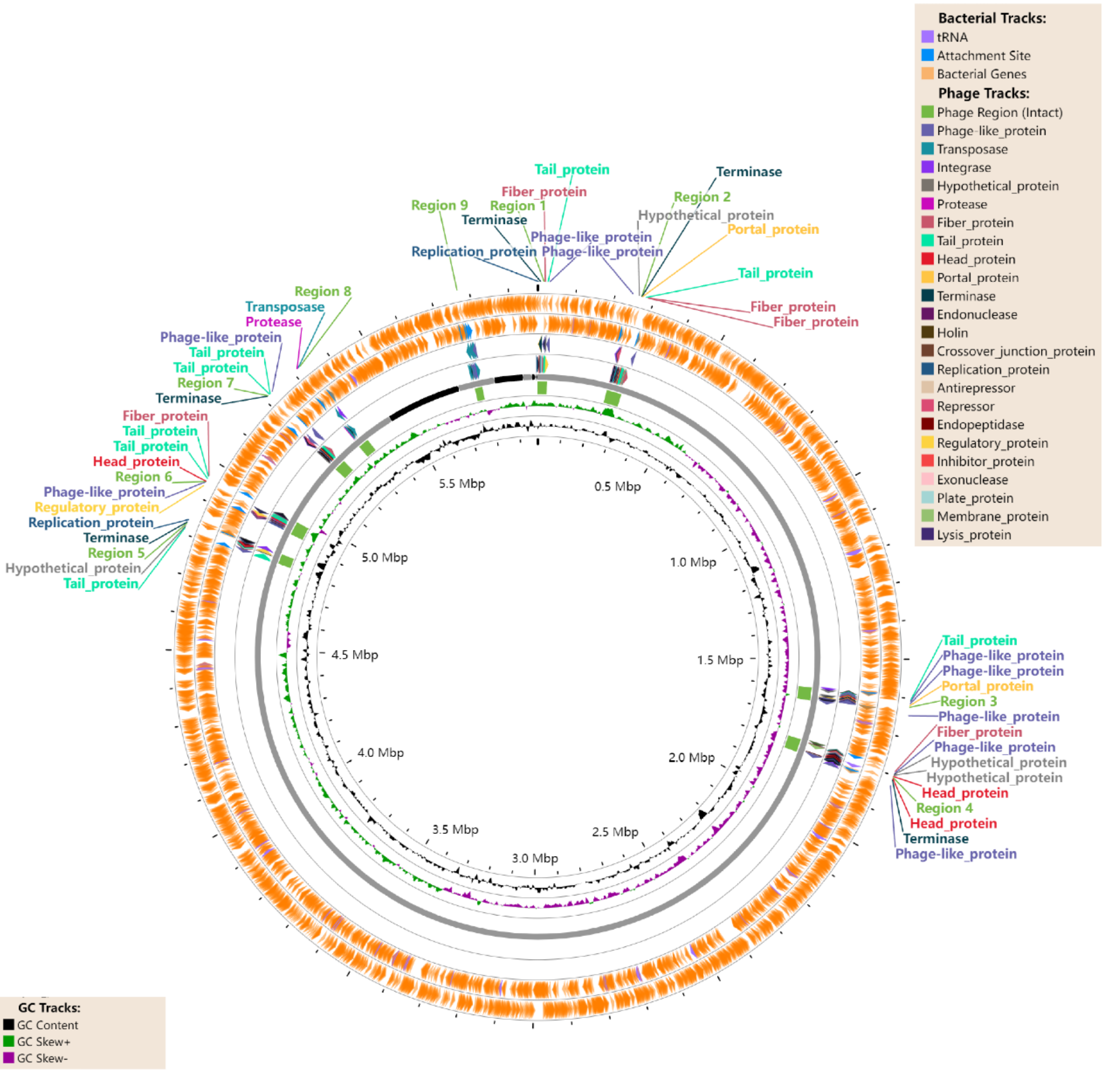

Fig S1 The annotated prophages in the genome of *Escherichia coli* ymmD45.

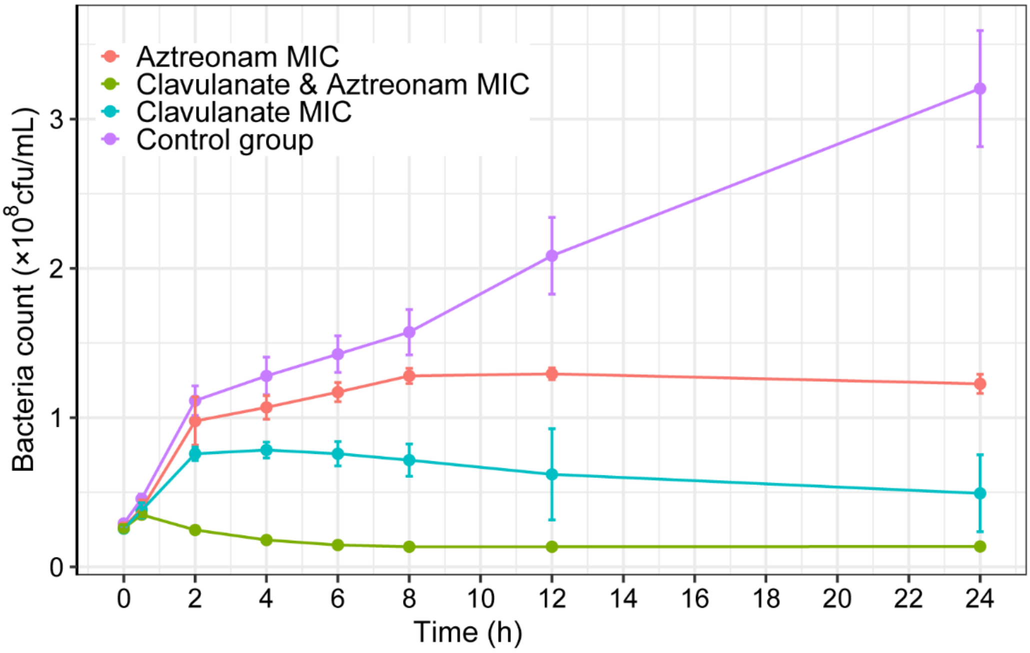

Fig S2 Growth curves of *Escherichia coli* ymmD45 exposed to 1×MIC aztreonam, 1×MIC clavulanate and 1×MIC clavulanate/aztreonam.

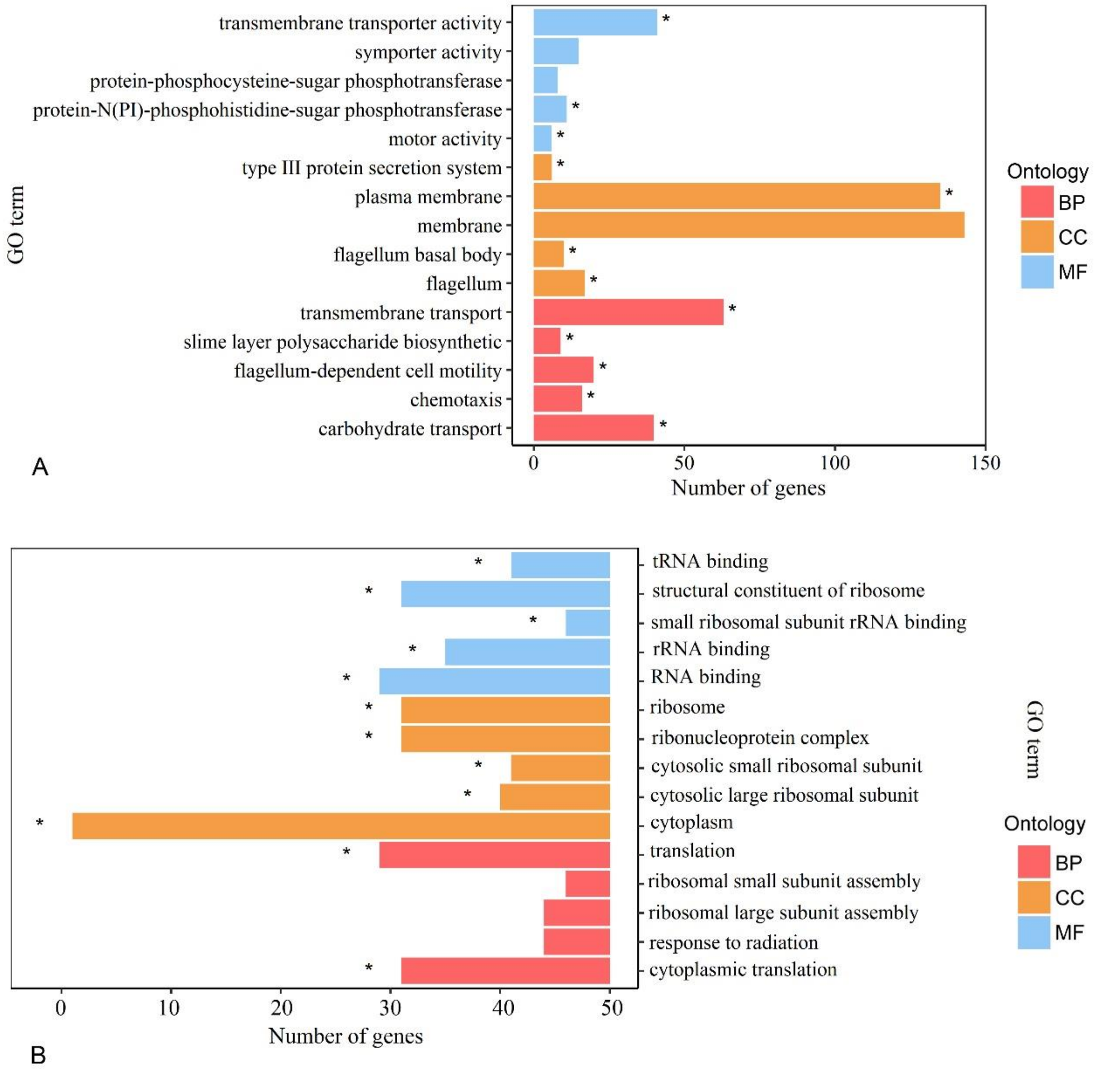

Fig S3 Differential gene expression and pathway enrichment analysis of 0.5 mg/L aztreonam exposure vs. the control. BP, CC and MF represent biological process, cellular component and molecular function, respectively. * $P < 0.001$. A - GO analysis of up-regulated genes. B - Pathway enrichment analysis on down-regulated genes.

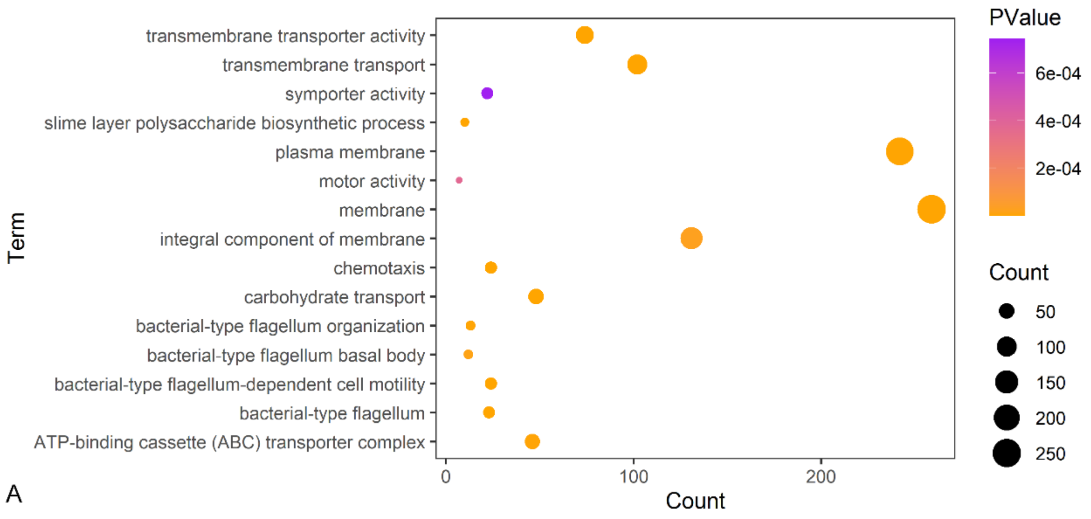

A

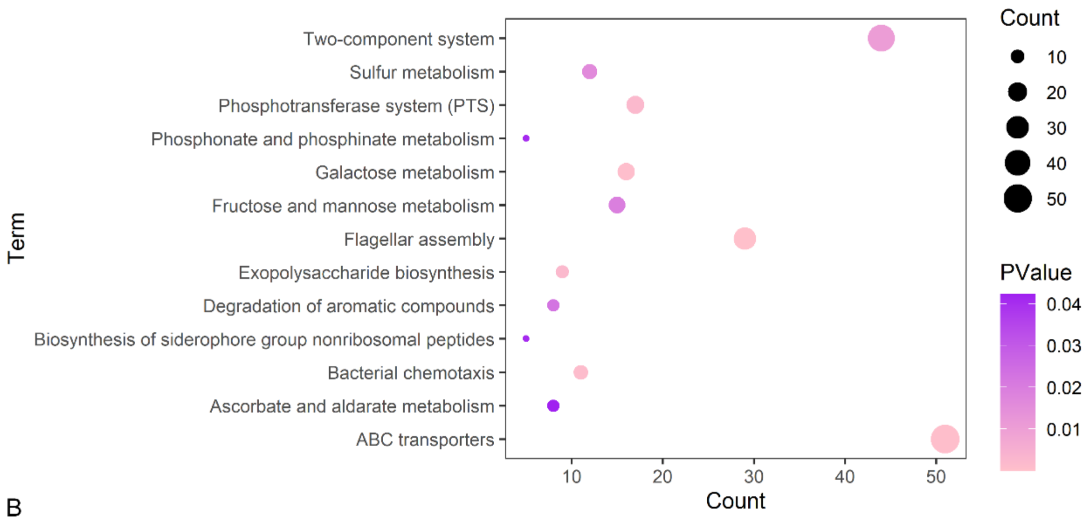

B

Fig S4 Differential gene expression and pathway enrichment analysis of 0.5 mg/L clavulanate exposure vs. control. A - GO analysis on up-regulated genes. B - KEGG pathway analysis on up-regulated genes. BP, CC and MF represent biological process, cellular component and molecular function, respectively.

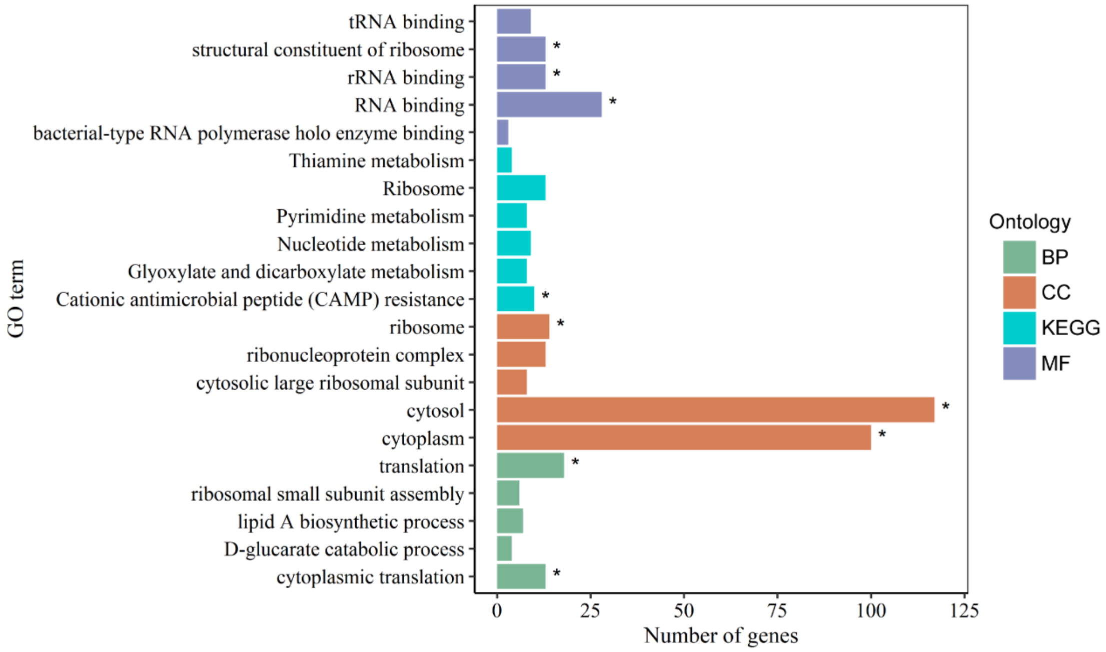

Fig S5 Down-regulated genes from GO and KEGG analysis following 0.5 mg/L clavulanate exposure vs the control. BP, CC and MF represent biological process, cellular component and molecular function, respectively. * $P < 0.001$.

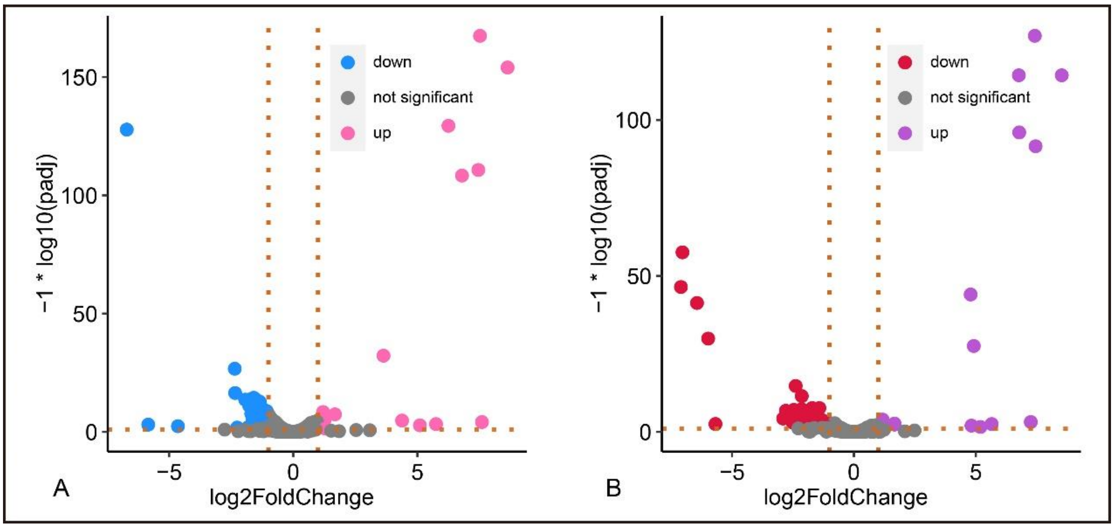

Fig S6 Differential gene expression. The dashed lines represent the threshold of extremely significantly up- or down-regulated genes (|log_2FoldChange|>1 and padj ≤ 0.05; padj is adjusted *P* value using Benjamin-Hochberg correction). A - Differential gene expression upon 0.5/0.5 mg/L aztreonam-clavulanate combination exposure vs. aztreonam exposure alone. Pink, blue and grey dots represent significantly up-regulated, down-regulated and not significantly differentially expressed genes, respectively. B - Differential gene expression upon 0.5/0.5 mg/L aztreonam-clavulanate combination exposure vs. clavulanate exposure alone. Purple, red and grey dots represent significantly up-regulated, down-regulated and not significantly differentially expressed genes, respectively.
